# Effect of probiotics on immune cells in young Japanese Black calves responding to vaccination against bacterial respiratory diseases

**DOI:** 10.2478/jvetres-2025-0013

**Published:** 2025-03-25

**Authors:** Shogo Takeda, Hiromichi Ohtsuka, Keigo Kosenda

**Affiliations:** Veterinary Medicine, Obihiro University of Agriculture, Obihiro-shi, Hokkaido, 080-8555, Japan; Hidaka C.L.C, Niikappu-cho, Hokkaido, 059-2403, Japan; Veterinary Medicine, Rakuno Gakuen University, Ebetsu-shi, Hokkaido, 069-8501, Japan

**Keywords:** probiotics, T cell, vaccination, bacterial respiratory disease, young Japanese Black calves

## Abstract

**Introduction:**

The vaccination against bacterial respiratory diseases in calves has been generally recognised as useful for the prevention of infections. Inducing an immunological response after vaccination is important for obtaining protection from infections. The aim of the study was to investigate the effects of probiotics on the immunological response to vaccination against bacterial respiratory diseases in young Japanese Black calves.

**Material and Methods:**

Twenty-four Japanese Black calves were randomly divided into two groups (12 calves for the research group and 12 calves for the control group) on the seventh day of life (dol). The research group received 30 g per day of live bacteria mix consisting of *Streptococcus faecalis, Clostridium butyricum* and *Bacillus mesentericus* until the 63^rd^ dol. The control group did not receive the bacteria mix. All calves were vaccinated against bacterial respiratory diseases twice, at 21 and 42 dol. Blood samples were obtained from all calves at 7, 21, 42 (prior to the second vaccination), 45, 49 and 63 dol for determination of antibody titres, leukocyte numbers and cytokine genes.

**Results:**

Lymphocyte counts, T cell (CD3^+^, CD4^+^ and CD8^+^ cell) counts and relative expressions of cytokine genes (interleukin (IL)-4 and IL-17A) at 45, 49 and 63 dol were significantly higher in the research group compared than in the control group.

**Conclusion:**

The addition of probiotics to young Japanese Black calves’ feed promoted an immunological reaction to vaccination against bacterial respiratory diseases.

## Introduction

There is a high incidence of respiratory diseases in young calves on beef cattle farms, and it causes significant economic losses (25). Most respiratory diseases in calves are caused by virus and bacterial infections, the latter often being with *Mycoplasma* genus and virus infevtions Pasteurellaceae family members. Among them, *Pasteurella multocida, Mannheimia haemolytica* and *Histophilus somni* are normal flora in the upper respiratory tracts of healthy calves, but are the main causes of respiratory diseases through opportunistic overgrowth (9). Vaccinations against these bacteria in calves have been generally recognised as useful for the prevention of bacterial respiratory diseases. Recent studies reported increased blood antibody titres after two administrations of vaccines against bacterial respiratory diseases in young calves (12, 19).

Inducing immunological response after vaccination is important for obtaining protection from infections. However, immune function is not fully developed in calves with lower numbers of activated effector cells, and it is hard to increase antibody titres after vaccination in these recipients (29). Calves of Japanese Black beef cattle originating from Japan are clear examples of animals with lower numbers of T cells and B cells than the ubiquitous Holstein calves (16). In addition, Japanese Black calves showed lower cytokine gene expressions when CD4, CD8 and γδ T cells were stimulated with mitogens. Thus they are considered to be at higher risk because of their lower immune functions (14).

Probiotics are live bacterial feed additives which benefit host animals by improving the balance of their gut microbiota (2). In humans, probiotics have been reported to protect against infections by improving immune function (20). Supplementation of probiotics also promoted specific antibody production against intestinal pathogens and oral vaccines (3). In sheep, probiotic supplements increased antibody titres and interleukin (IL)-10 and IL-17A cytokine gene levels in mononuclear cells after vaccination against bovine herpesvirus (22). In cattle, probiotics are thought to promote growth and improve digestive function (27). Some preventive effects of probiotics against digestive tract diseases in vulnerable young calves have also been reported (8). The effects of probiotics on immune functions, and especially after vaccination, have not been elucidated in Japanese Black calves. In this study, we investigated the effects of probiotics on immune cells after vaccination against bacterial respiratory diseases in young calves of this breed, which are known to have inferior immune functions compared to Holstein calves.

## Material and Methods

Twenty-four Japanese Black calves kept on one farm in Hokkaido were used. All calves stayed with their dams until the seventh day of life (dol), and were then transferred to individual hutches for feeding with milk replacer. On the seventh dol, calves were randomly divided into two groups: the research group (n = 12) and the control group (n = 12). The research group received 30 g per day of a commercial probiotic mixture with *Streptococcus faecalis, Clostridium butyricum* and *Bacillus mesentericus* (Bio-Three for animal, Toa Animal Health, Tokyo, Japan) from 7 to 63 dol, added to the milk replacer. The control group did not receive this supplement.

Each of the experimental calves received a 1 mL intramuscular injection of the combined inactivated vaccine for *P. multocida, M. haemolytica* and *H. somni* (Cattlebact 3; Kyotobiken, Kyoto, Japan) following the procedure of Mori *et al*. (12).

The amount and nutritional composition of feed during the experimental period are shown in Table 1. All experimental calves met the nutritional requirements based on the Japanese Feeding Standard (the Japanese feeding standard for beef cattle, 2008 version, Japan Livestock Industry Association, Tokyo). In addition, all experimental animals were handled in compliance with the “Guidelines for Proper Conduct of Animal Experiments” published by the Science Council of Japan. All animals remained clinically healthy and no animal required medical treatment throughout the experiment. The procedures used in the present study were in accordance with the principles and guidelines for animal use set by the Animal Experiment and Care Committee of Obihiro University of Agriculture and Veterinary Medicine, Obihiro, Japan (Permit No. 24-85).

**Table 1.** Nutrient composition of diets in the Japanese Black calves

Dietary component	Percentage of dry matter intake from commercial feed diet	
	Milk replacer	Starter
TDN	116	75
CP	25	20
Fat	25	2
NDF	1	10

1 TDN – toral digestible nutrients; CP – crude protein; NDF – neutral detergent fiber

Blood samples were obtained at 7, 21, 42, 45, 49 and 63 dol from the jugular vein, and serum samples were isolated by centrifuging immediately after sampling. They were stored at –30°C until analysis.

### Measurement of antibody titres

Serum antibodies against *P. multocida, M. haemolytica* and *H. somni* were detected using an ELISA as previously described (13).

#### Analysis of the expression of leukocyte surface antigens

A flow cytometer (Beckman Coulter, Brea, CA, USA) was used with an indirect fluorescent antibody technique for detection and analysis. All but one primary antibodies used were specifically against bovine and were anti-CD3 (MMIA; pan T cell), anti-CD4 (CACT183B; helper T cell), anti-CD8 (CACT80C; killer T cell), anti-CD21 (GB25A; B cell), TcR1-N12 (CACT61A; γδ T cell) and MHC class II (TH14B; B cell and monocyte) (all from VMRD, Pullman, WA, USA). The final primary antibody was against human CD14 (MY-4; monocyte) (Beckman Coulter). Calculations of absolute numbers of those subpopulations were performed using percentages of mononuclear cells and granulocytes in the cytogram obtained from the analysis of cell surface antigen expression, the percentage of antigen positive cells and the leukocyte counts using the formula below:

Absolute count of marker positive cells (×10^2^/μL) = marker positive cell percentage within mononuclear cells × leukocyte count × percentage of mononuclear cells within total leukocytes.

#### Real-time PCR

Messenger RNA (mRNA) was extracted from the mononuclear cells isolated from heparinised blood using density gradient centrifugation. Referring to previous reports (17, 21), the complementary DNA used for the real time PCR was synthesised using extracted mRNA. The PCR was performed using the designed bovine primers shown in Table 2. Beta actin was used as the internal gene control. Following the protocol provided by the manufacturer of the SYBR Green PCR Master Mix (Applied Biosystems, Foster City, CA, USA), the real-time PCR was performed on an ABI Prism 7300 Sequence Detector (Applied Biosystems). The gene expression level for each cytokine was expressed as a relative value of the threshold cycle (Ct value) for amplification.

**Table 2.** Primers used for real-time PCR expression analysis

Gene	GenBank Accession No.	Product length (base pairs)	Primer	5′–3′ sequence
IL-4	NM_173921	117	Forward	GCCCCAAAGAACACAACTGA
			Reverse	GAGATTCCTGTCAAGTCCGC
IL-12p40	NM_174356.1	101	Forward	CACCCCGCATTCCTACTTCT
			Reverse	TGACTTTGGCTGAGGTTTGG
IL-17A	NM_001008412.2	138	Forward	ATCTCACAGCGAGCACAAGT
			Reverse	GTGGGATGATGACTCCTGCC
IFN-γ	NM_174086	108	Forward	TCAAATTCCGGTGGATGATCT
			Reverse	CTTCTCTTCCGCTTTCTGAGG
TGF-β	NM_001166068.1	140	Forward	TGACCCGCAGAGAGGAAATAG
			Reverse	GTTCATGCCGTGAATGGTG
β-actin	NM_173979.3	76	Forward	TCTTCCATAGGACACAATGCC
			Reverse	GAGGCATACAGGGACAGCAC

1 IL – interleukin; IFN – interferon; TGF – transforming growth factor

All data are expressed as mean ± standard error and the MannWhitney U test was used for statistical analysis to reveal statistically significant differences (P-value < 0.05) between groups on the same days of life.

## Results

**Fig. 1.**
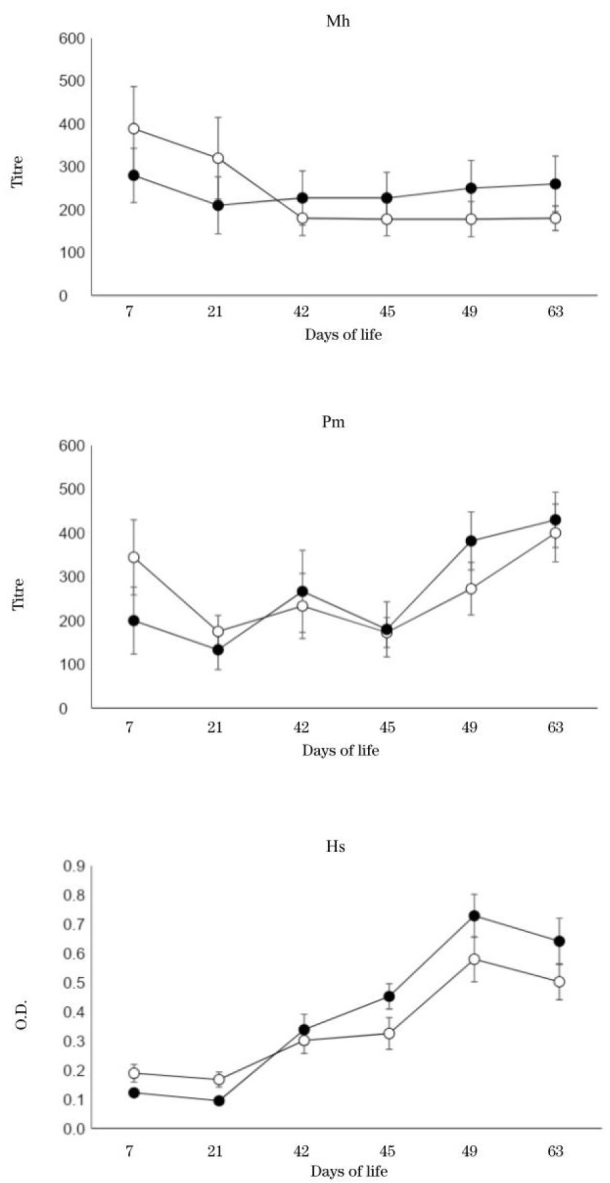
Changes in antibody titres against *Pasteurella multocida, Mannheimia haemolytica* and *Histophilus somni*. Solid circles – research group; hollow circles – control group. Results are expressed as the mean ± standard error (n = 24). O.D. – optimal density

Changes in serum antibody titres in the research and the control groups are shown in Fig. 1. Antibodies against *M. haemolytica* were higher in the research group than the control group at 42, 45, 49 and 63 dol. Antibodies against *P. multocida* were higher in the research group than the control group at 49 dol. Although antibody titres against *H. somni* were higher in the research group than the control group at 45, 49, and 63 dol, the differences from the control group were not statistically significant throughout the observation period.

**Fig. 2.**
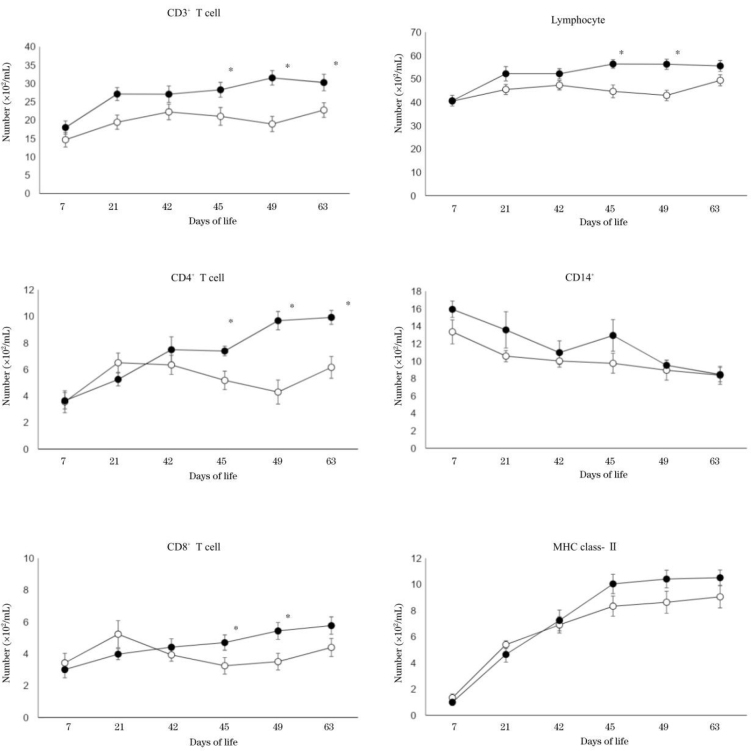
Changes in numbers of peripheral lymphocyte, CD3^+^, CD4^+^, CD8^+^, CD14^+^ and MHC class-II cells. Solid circles – research group; hollow circles – control group. *–significant difference (P-value < 0.05) between two groups on the same day of life

Changes in peripheral blood immune cell numbers in both groups are shown in Fig. 2. The numbers of lymphocytes after the second vaccination were significantly higher in the research group than the control group at 45 and 49 dol (P-value < 0.05). The numbers of CD3^+^ T cells and CD4^+^ T cells in the research group were significantly higher after the second vaccination at 45, 49 and 63 dol (P-value < 0.05), as were the numbers of CD8^+^ T cells in the research group after the second vaccination at 45 and 49 dol (P-value < 0.05). Also, the numbers of CD14^+^ cells in the research group were higher than those in the control group at 45 dol (after the second vaccination); however, the difference was not statistically significant. Although the numbers of MHC class-II cells in the research group were higher at 45, 49 and 63 days of age, they were not statistically significant compared to the numbers in the control group.

**Fig. 3.**
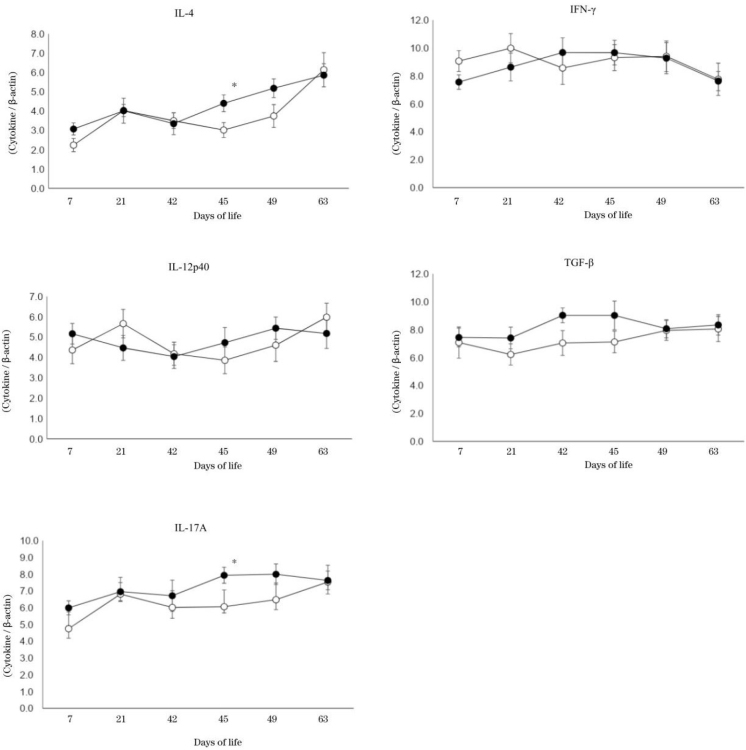
Changes in relative gene expressions of interleukin (IL)-4, IL-12p40, IL-17A, interferon (IFN)-γ and transforming growth factor (TGF)-β. Solid circles – research group; hollow circles – control group. *–significant difference (P-value < 0.05) between two groups on the same day of life

The changes in relative gene expressions of cytokine mRNA inside the peripheral blood mononuclear cells are shown in Fig. 3.

The relative gene expressions of IL-4 and IL-17A in the research group after the second vaccination were significantly higher than those in the control group at 45 dol (P-value < 0.05). However, IL-12p40, IFN-γ and TGF-β gene expressions did not show significant differences between groups.

## Discussion

This study investigated the effect of probiotics on immune cells after vaccination against bacterial respiratory diseases in young Japanese Black calves. The antibody titres against all three bacteria included in the vaccine were higher in the research group than in the control group after the second vaccination. Mori *et al*. (12) showed the booster effects (increases in antibody titres) of the same vaccine as we used for this study in Holstein calves when the second 1 mL vaccination was given two to four weeks after the first vaccination of the same volume at 5–12 dol. Otomaru *et al*. (19) vaccinated Japanese Black calves with 2 mL of the same vaccine as our experiment at one and four weeks of age, and observed a significantly lower incidence of respiratory diseases and significantly higher antibody titres against all three bacteria contained in the vaccine compared to the disease incidence and antibody titres in nonvaccinated calves. These results indicated that double administration of this inactivated bacterial vaccine was required for sufficient responses (1). In our current study, both groups showed increased antibody titres after the second vaccination, and the antibody titres were similar to those noted in the previous studies with the same vaccine in Holstein calves as well as Japanese Black calves.

There has been no report regarding the effects of probiotics on peripheral blood immune activation in Japanese Black calves. In humans, T-cell activation by antigen-presenting cells is important for enhancement of immune function (10), and involvement of intestinal microbiota in the whole-body immune function has been reported (26). This led to the usefulness of providing probiotics in feed to young animals in enhancing immune function and T cell activity having been studied (11). For example, experimental feeding of probiotics to germ-free mice, of which the lymph node is about 1/2 of its weight in conventional mice and in which the development of peripheral lymphatic tissue is extremely poor, was observed to enhance T-cell activation by intestinal microbiota and increase immune responses (7). In addition, antibody production was induced when B cells contacted antigens in the mucosal tissue (4). Further, increased immune responses to vaccination as the effect of probiotics in animals and humans have been reported (22, 23).

Immediately after birth, the number of lymphocytes and the activities of cellular as well as humoral immune functions in calves are low, and they gradually increase as calves grow (18). In our current study, the numbers of CD3^+^, CD4^+^ and CD8^+^ T cells in the peripheral blood significantly increased after the second vaccination in the research group. This suggested that propagation of peripheral T cells was induced as the booster effect in the research group. Even in the Japanese Black calves, which are known to have inferior immune function, probiotics might have activated intestinal mucosa immune tissue and increased T-cell propagation in response to vaccination.

In younger calves, helper T (Th)1 and Th2 type lymphocytes have lower responsiveness than they do in adult cattle (15). In our current study, relative gene expression values of IL-4 and IL17A in the mononuclear cells increased after the second vaccination in the research group. Among helper T cells, the Th2 type produces IL-4, and the Th17 type produces IL-17A. Interleukin 4 assists humoral immunity as well as T cell differentiation and proliferation (6). Interleukin 17 is a cytokine produced by activated T cells, and it promotes the activities of neutrophils which have the purposes of directly attacking invading bacteria and defending hosts (28). A previous report described how the Th17 prevalence in CD4^+^ T cells rose fourfold in Bacillus Calmette-Guérin–vaccinated neonates compared to unvaccinated neonates (24). In addition to having lower response from Th1 and Th2 cells, young calves also have insufficient antibody production by B cells (5), which means that their antibody production after vaccination is poor compared to that of adult cattle (30). In spite of the increase in IL-4, which is related to humoral immunity, in the research group after the second vaccination in our current study, there were no changes in B cell numbers in either group after vaccination. It suggests that even if T cells were activated by vaccination in young calves after vaccination, humoral immunity might have still been insufficiently mature for any enhancing effect on the production and responsiveness of B cells to be manifest.

## Conclusion

This study showed the possibility that probiotics improved and stabilised the intestinal environment, which might have fortified immune cells inside the intestinal mucosa. Degrees of enhanced response of phagocytes vary depending on the types, doses and survival of probiotics (5). In our current study, it is not clear what kind of mode of action by probiotics produced our results, and it should be studied in future.

Japanese Black calves have lower numbers of monocytes, T cells and B cells compared to Holstein calves (16), and this might be the reason for lower immune responsiveness in Japanese Black calves. In our current study, increased lymphocyte numbers were observed as the immune response to vaccination in the research group. Although this study used a limited number of calves, it suggested the usefulness of probiotic supplementation in young Japanese Black calves’ feed in building an effective vaccination programme and managing diseases with an effective set of strategies.
